# The Accurate Prediction of Antibody Deamidations by Combining High-Throughput Automated Peptide Mapping and Protein Language Model-Based Deep Learning

**DOI:** 10.3390/antib13030074

**Published:** 2024-09-10

**Authors:** Ben Niu, Benjamin Lee, Lili Wang, Wen Chen, Jeffrey Johnson

**Affiliations:** 1 Discovery Biotherapeutics, Bristol Myers Squibb, San Diego, CA 92121, USA; 2 Department of Molecular and Cell Biology, University of California, Berkeley, CA 94720, USA

**Keywords:** automation, peptide mapping, deamidation, deep learning, protein language model

## Abstract

Therapeutic antibodies such as monoclonal antibodies (mAbs), bispecific and multispecific antibodies are pivotal in therapeutic protein development and have transformed disease treatments across various therapeutic areas. The integrity of therapeutic antibodies, however, is compromised by sequence liabilities, notably deamidation, where asparagine (N) and glutamine (Q) residues undergo chemical degradations. Deamidation negatively impacts the efficacy, stability, and safety of diverse classes of antibodies, thus necessitating the critical need for the early and accurate identification of vulnerable sites. In this article, a comprehensive antibody deamidation-specific dataset (n = 2285) of varied modalities was created by using high-throughput automated peptide mapping followed by supervised machine learning to predict the deamidation propensities, as well as the extents, throughout the entire antibody sequences. We propose a novel chimeric deep learning model, integrating protein language model (pLM)-derived embeddings with local sequence information for enhanced deamidation predictions. Remarkably, this model requires only sequence inputs, eliminating the need for laborious feature engineering. Our approach demonstrates state-of-the-art performance, offering a streamlined workflow for high-throughput automated peptide mapping and deamidation prediction, with the potential of broader applicability to other antibody sequence liabilities.

## 1. Introduction

Monoclonal antibodies (mAbs) represent one of the predominant classes of therapeutic proteins; recently, more complex formats of antibodies, such as bispecific and multispecific antibodies, and fusion proteins have debuted to treat various diseases in multiple different therapeutic areas [1,2,3,4]. These therapeutic antibodies are engineered to bind selectively to their target antigens, modulating the biological pathways to achieve the therapeutic effects. However, during the development, manufacturing, and storage of the therapeutic antibodies, various sequence liabilities may arise, potentially impacting their safety, efficacy, and stability. An antibody sequence liability refers to the specific antibody amino acid residues (namely, hot spots) undergoing chemical degradations, structural alterations, or enzymatic modifications [5,6]. One of the most common and putatively most concerning of the sequence liabilities of the therapeutic antibodies is deamidation, a spontaneous chemical process particularly involving asparagine (N) and glutamine (Q) residues, converting them into negatively charged aspartate (D) and glutamate (E) residues, respectively, through several possible non-enzymatic pathways [7]. Deamidation has been reported to compromise both *in vivo* and *in vitro* biological activities, structural integrity, pharmacokinetics, antigen-binding affinity, and even the immunogenicity of diverse classes of antibodies [5,7,8]. Therefore, identifying the liable sites for deamidations has become a critical step. 

In particular, during the drug discovery phase, early access to antibody deamidation liabilities is beneficial to de-risk the drug candidate selection process and accelerate drug development. Typically, forced degradations by thermal and high pH stresses have been employed to enrich the liable deamidated residues prior to any experimental measurements [9]. Nevertheless, measuring deamidation, in particular, by assessing site-specific deamidation information is challenging because (i) conventional reversed phase separation techniques or charge-based separation methods (e.g., ion exchange chromatography and capillary isoelectric focusing (cIEF)) lack the specificity to resolve interfering species that co-elute or to localize deamidation at the amino acid level [10,11]; and (ii) intact or reduced mass analysis cannot unambiguously detect deamidation owing to the small +0.98 Da mass shift that easily falls in the assay variability [12]. The LC-MS/MS-based peptide mapping method, which enzymatically dissociates the protein into smaller peptide pieces, spatially separating those peptides followed by high-resolution MS detection, on the other hand, can confidently detect, quantify, and localize the deamidations, providing site-specific deamidation information [13,14]. Nevertheless, peptide mapping is intrinsically labor-intensive in both the sample preparation and data processing. In addition, to accommodate the forced degradation followed by the peptide mapping of the sample preparations, the amount of purified antibody to initiate this task can be quite high. However, often, especially at the earlier stages, the experimental assessment of deamidations via forced degradation and peptide mapping is constrained by both the low availability of purified antibody material and the high demand of FTE/instrument resources. Given these limitations, computational tools have become increasingly common for early antibody deamidation profiling, because they eliminate the need for sample consumption and afford a fast turnaround and potentially high throughput while requiring minimal cost [15,16,17,18].

Computational models for predicting deamidation occurrences have been around for decades and have been undergoing continued evolvement [17,18,19,20,21,22,23,24,25]. Informed by prior knowledge that flanking sequences, secondary and tertiary structures, solvent accessibility, and structural rigidity can all impact antibody deamidations, these models can be largely divided into two categories, namely, sequence-based models and structure-based models. For example, to date, some sequence-based deamidation tools simply designate all deamidation “hot spots” based on the sequence motifs of NG and NS, determined from a model that used pentapeptide surrogates to probe various deamidation rates [26]; however, in reality even the same sequence motifs are likely to exhibit varied deamidation rates due to changes in solvent accessibility and the high order structure. In general, conventional sequence-based models are typically easy to use but suffer with respect to the accuracy of their performance. By comparison, structure-based models typically perform better, as additional descriptors, such as the secondary structure, tertiary structure, SASA, backbone, and side-chain dihedral angles, are taken into account. For example, in separate studies, Jia et al. [21] and Delmar et al. [19] developed machine learning models for liable Asn deamidation prediction by mining the structural parameters such as the backbone nucleophilic attack distance, dihedral angles, side-chain dihedral angles, torsion angles, etc., from crystal structures and 3D antibody structural homology models, respectively. Hoffmann et al. [20] recently reported an effective QSAR model, factoring in the accessible surface area (ASA) of the residue, the pKa value of the backbone amide, and the root mean square deviations of both the alpha carbon and the side chain. As one would expect, different structure-based models may require the different crafting and selection of structural features. Despite these successes in structure-based approaches, a prerequisite to enable the use of these models is either an experimental crystal structure or an *in silico* structural homology model; in some cases, molecular dynamic (MD) simulations were also required in order to compensate for flexible loop conformations [25]. This has limited the ease of access to structure-based approaches. In addition, for more complex modalities such as multispecific or fusion proteins, it is inherently challenging to even obtain structural homologies. 

With the recent advancement of artificial intelligence and natural language processing techniques, pretrained protein language models (pLMs) employing transformer architectures [27] have debuted as an increasingly widespread paradigm to extract contextual information directly from sequences, enabling the effective amino acid-level representations of various proteins, including therapeutic antibodies. Notable examples of pLMs include ProtBert [28], ESM-1b [29], ESM-2 [30], and ProtT5 [31]. These models were pretrained on massive sets of protein sequences in an unsupervised manner via the objectives of masked language modeling and were shown to be capable of learning complex contextual dependencies among residue properties and structural features. These learned representations of proteins, often manifested as vectors (also known as embeddings), are meaningful “heuristic features” about proteins, potentially eliminating the need for structural homology, feature engineering, evolutionary searches, and multiple sequence alignments (MSAs); all of these processes are typically time consuming and computationally expensive. Indeed, by simply taking protein sequences as the input, pLM embeddings can be used in a broad range of tasks including protein 3D structure prediction [30,32,33], subcellular localization [34], mutational impact prediction [35], and, more recently, post-translational modifications [36,37].

In this study, we propose the use of embeddings from a state-of-the-art pLM, namely, ESM-2, for the prediction of deamidation sites in therapeutic antibodies. The use of a pLM has demonstrated a trail of success in predicting various domain-specific tasks. However, to our best knowledge, the feasibility of using a pLM for deamidation predictions is yet to have been explored. We present a novel, chimeric deep learning model that integrates the contextual residue-level embeddings with the local amino acid sequence information. Notably, we show that this method achieves state-of-the-art performance by directly taking antibody sequences as the input with no requirement for handcrafted or manual feature extractions. In addition, the method can also project the quantitative deamidation extents at future time points. Last but not least, we underscore that the presented systematic workflow, involving high-throughput automated peptide mapping followed by a pLM-fueled deep learning framework, is applicable to other sequence liabilities of therapeutic antibodies.

## 2. Materials and Methods

### 2.1. Chemicals and Reagents

All antibodies described in this work, except for the NISTmAb antibody, were produced using Chinese hamster ovary (CHO) cell lines at Bristol Myers Squibb. The NISTmAb, which is a humanized IgG1 monoclonal antibody, was obtained from Sigma-Aldrich (cat. NIST8671, St. Louis, MO, USA). The trypsin was purchased from Promega (cat. V5280, Madison, WI, USA) and the guanidine hydrochloride (8.0 M, Cat. 24115) and microdialysis cassettes (cat. 88260) were purchased from Thermo Fisher Scientific (Waltham, MA, USA). All the other reagents were purchased from Sigma-Aldrich (St. Louis, MO, USA). 

### 2.2. Accelerated Thermal Stress 

**Figure 1:**
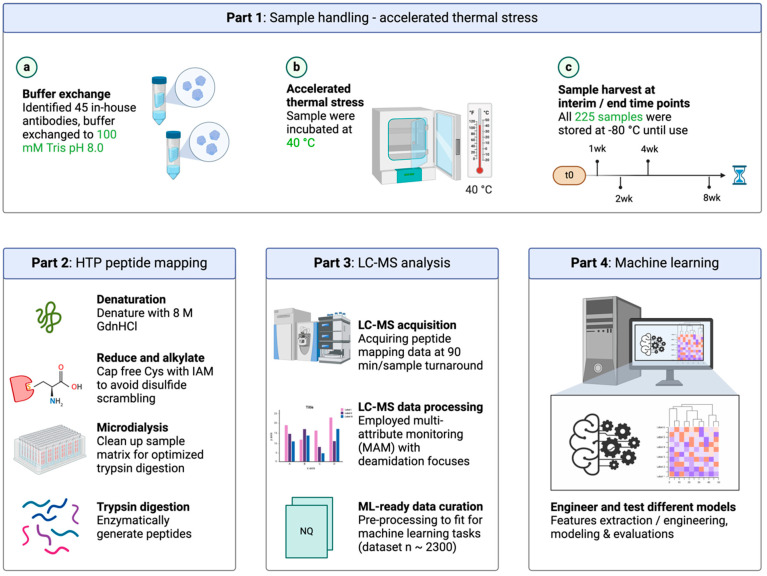
Development of a systematic workflow to predict therapeutic antibody deamidations. Starting from forced degradation at 40 °C at a pH of 8.0 for 8 weeks with interim time points (t0, 1 week, 2 weeks, 4 weeks, and 8 weeks), antibody samples were subjected to high-throughput automated peptide mapping followed by LC-MS/MS analysis. Machine learning models were trained on a curated, deamidation site-specific dataset.

A panel of 51 antibodies, including NISTmAb and 50 in-house antibodies of varied modalities, including monoclonal antibody, bispecific antibody, and fusion protein, were buffer-exchanged to 100 mM of Tris at a pH of 8.0 at 5.0 mg/mL, followed by incubation at 40 °C for up to 8 weeks with interim time points after 1 week, 2 weeks, and 4 weeks (Figure 1). The samples were stored at −80 °C upon due time. For the t = 0 control, the samples were put in storage immediately after the buffer exchange. This gave 255 samples overall; these samples were then subjected to the peptide mapping protocol and LC-MS/MS analysis.

### 2.3. Automated Peptide Mapping

A high-throughput fully automated peptide mapping sample preparation platform was developed by using the Lynx liquid handling robotic system (Dynamic Devices, Wilmington, DE, USA). The robot was equipped with a plate gripper and an individually addressable 96-channel pipetting arm; each channel had a maximum capacity of 1250 μL. The plate gripper enabled the 96-well plate movements on the deck upon the method initiation. The liquid handler deck was equipped with a BioShake Q1 (Q Instruments, Jena, Germany) that enabled the heating, cooling, and shaking required in the protocol. 

The detailed sample handling steps are described in the Supplemental Information. Briefly, the Lynx robotic system performed the following procedures sequentially: the protein sample concentration normalization, denaturation, disulfide bond reduction, free cysteine alkylation, microdialysis-based buffer exchange, trypsin digestion, quenching of the reaction, and cooling storage. Upon completion, the resulting plate containing the quenched digests covered with a light protective lid was placed on the cooling block until retrieval by the analyst. 

### 2.4. LC-MS/MS Analysis 

The Vanquish UHPLC module (Thermo Fisher Scientific) was configured to directly take the resulting 96-well plate from the automated peptide mapping protocol. An aliquot of peptide digests (6 μg) was loaded onto a reversed phase C18 column (130 Å, 1.7 μm, 2.1 × 150 mm; Waters, Milford, MA, USA) and spatially separated using a linear gradient from 0% to 40% in the mobile phase B, consisting of 0.02% (*v*/*v*) of TFA in acetonitrile at a flow rate of 0.2 mL/min. The column temperature was maintained at 55 °C. The detection was performed using an Exploris 480 mass spectrometer (Thermo Fisher Scientific), with an electrospray ionization source operated in positive polarity at a spray voltage of 3.5 kV and a capillary temperature of 320 °C. The mass range of the precursor ions was set at 250–2000 *m/z* with a high resolving power of 120,000. The data acquisition was performed in the top 5 data-dependent acquisition mode, with the dynamic exclusion duration set for 5 s after each scan, in an attempt to further boost the MS/MS spectra even for lower abundance species, facilitating site-specific modification assignment during the data analysis. The details regarding the database searching and post-translational modification (PTM) identification and quantification are described in the Supplemental Information.

## 3. Results

### 3.1. High-Throughput (HTP) Automated Peptide Mapping

**Figure 2:**
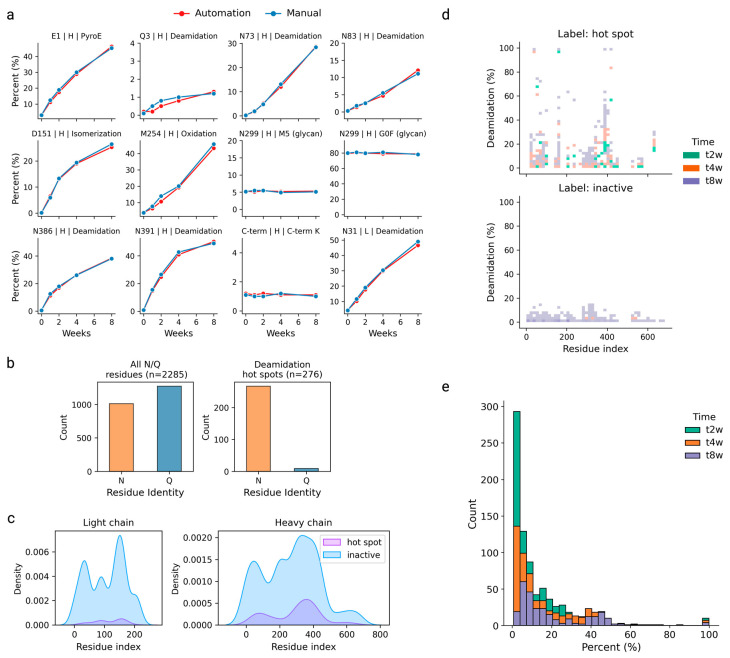
(**a**) The quantitation of the PTMs of various categories by automated peptide mapping (red) for stressed mAb-A samples at five different time points (t = 0, 1, 2, 4, and 8 weeks); the same set of samples were subjected to manual peptide mapping (blue) to demonstrate a good quantitation comparability between the two protocols. (**b**) Bar graphs illustrating the imbalanced nature of the final deamidation-specific training dataset (n = 2285). (**c**) The overall distribution of deamidation binary labels (hot spot versus inactive) in the dataset along light-chain and heavy-chain sequences, respectively. (**d**) The overall distribution of quantitative deamidation extents at t = 2-week, t = 4-week, and t = 8-week time points compared with respect to the deamidation labels, indicating that the sites labeled as hot spots gave a broader deamidation distribution and higher extents, whereas sites labeled as inactive showed a narrower distribution centered at lower (<20%) deamidation extents. (**e**) A histogram of the total quantitative deamidation in the dataset by three time points (t = 2, 4, and 8 weeks), showing that the deamidation extents shift towards higher percentages over the time course.

The peptide mapping-rooted approach, recently dubbed the multi-attribute method (MAM), employs mass spectrometry detection for the simultaneous identification and quantitation of many protein quality attributes including deamidations [13,14,38,39]. Although peptide mapping is ideal in providing site-specific modification readouts, the throughput of peptide mapping has long been a bottleneck limiting its practicality especially in the drug discovery phase where the sample numbers are large and the expected throughputs are high. To address this, we developed an automated peptide mapping sample preparation protocol using the Lynx liquid handling system. The fully walk-off system processes a 96-well plate in 7 h with a high degree of reproducibility (Figure S1). The sample preparations for all the degradation samples included in this study (255 samples for a total of 51 antibodies; refer to the Material and Methods Section for details) were completed in 3 days using the described high-throughput peptide mapping platform (Figure 1). To illustrate the method reproducibility, we show in Table S1 the PTM% comparisons among the samples located at diagonal positions on a 96-well plate (A1 to H8). These site-specific PTM percentages demonstrated good repeatability in terms of quantification across a wide dynamic range (from 0.1% to 100%). Note that in addition to detecting deamidations, the method can simultaneously measure and quantify a number of other PTMs including oxidation, isomerization, N-/C-terminal modifications, succinimide formation, and glycosylation (Figure 2a and Table S1). The levels of heavy-chain PENNY peptide deamidations (i.e., N387 and N392) were less than 2% and the Met255 oxidation was ~3% (Table S1), suggesting that our method did not generate artifact PTMs. 

Another important metric evaluating an automated procedure is its comparability to a manual procedure. We show that the automated procedure can be implemented interchangeably with its manual protocol equivalent, as the tryptic digestion profiles generated from the automation platform notably resembled those from the manual workflow (Figure S2). Furthermore, the PTM% obtained using the automated peptide mapping were comparable to those obtained using the manual protocol. Take mAb-A as example, where the samples were stressed at various time points (at 40 °C at a pH of 8.0) before submitting to automated and manual peptide mapping (Figure 2a): the outcome demonstrated great comparability for numerous PTMs, including deamidation, between the two protocols over a wide quantitation range from 0.1% to 80%. Overall, the developed high-throughput automated peptide mapping workflow significantly streamlined and expedited the sample processing, generating a large amount of high-quality data at the amino acid level ready for the subsequent machine learning endeavors.

Next, the processed peptide mapping data were further curated to concentrate on the deamidation outcome. Essentially, each deamidation instance was manifested as a site-specific, time-dependent profile consisting of five time points at t = 0, 1, 2, 4, and 8 weeks (for instance, the heavy-chain Q3, N73, N83, N386, and N391 deamidations in Figure 2a). In supervised machine learning, with the goal of classifying the deamidation sites into an active set (or hot spot) versus an inactive set (not liable or low risk), it is imperative to carefully label the dataset instances. In this study, each deamidation site was labeled by setting a fixed deamidation threshold. Specifically, for any site of interest (either N or Q residues), it was labeled as an active set if the increment of the measured deamidation extents from either the t_0_ to t_1week_ or from t_1week_ to t_2week_ time points exceeded 1.0%. Any remaining deamidation instances were labeled as inactive sites; these also included any N/Q residues that did not give measurable deamidations during the peptide mapping analysis. Of note, the LOQ in our peptide mapping assay was approximately 0.1%. For the training and test dataset split, we allocated a full dataset corresponding to 45 in-house antibodies as the training set and the remainder, including NISTmAb, as the independent test set.

The harvested training dataset revealed a pronounced imbalance, with 2285 labeled deamidation instances, predominantly skewed towards negative labels. Specifically, 276 instances were designated as deamidation hot spots, while 2009 were classified as inactive (Figure 2b). Notably, the distribution of deamidation hot spots was not confined to specific regions along the protein sequences; instead, they were observed to span across both the light and heavy chains (Figure 2c). Each deamidation instance in the dataset was accompanied by a binary label indicating its deamidation status, along with the experimental quantitative measurements of deamidation extents at t_2week_, t_4week_, and t_8week_. As illustrated in Figure 2d and Figure S3, the sites labeled as inactive exhibited consistently lower levels of deamidation compared to those identified as hot spots. The distribution of quantitative deamidation extents also showed a notable shift towards higher percentages over the course of the experiment (Figure 2e), corresponding to the gradually elevating deamidation extents from t_2week_ to t_8week_.

### 3.2. The Use of ESM-2 Embedding for Deamidation Site Prediction

Our objective was to construct models capable of classifying deamidation directly as active (indicating a hot spot or potential liability) or inactive (representing a low risk) for any site of interest (N or Q residues) within the antibody sequence, using only the antibody sequences themselves as the input. To achieve this, it was essential to first encode the antibody sequences into suitable representations prior to passing to downstream learning tasks. 

Among the various encoding schemes that extract vector representations (embeddings) directly from protein sequences [40], pretrained protein language models (pLMs) have emerged as particularly powerful tools. In our study, we employed protein language models to render latent, context-dependent embeddings. Specifically, the embeddings utilized in our work were derived from a pretrained ESM-2 model, which was trained on approximately 65 million unique protein sequences sourced from the UniRef [41] protein sequence database [30]. Of the many different sizes of pretrained ESM-2 models, which differ by the number of parameters ranging from eight million to 15 billion, we selected the one with 33 layers and 650 million learnable parameters (esm2_t33_650m_UR50D), striking a balance between the model performance, protein embedding sizes, and hardware constraints. 

**Figure 3:**
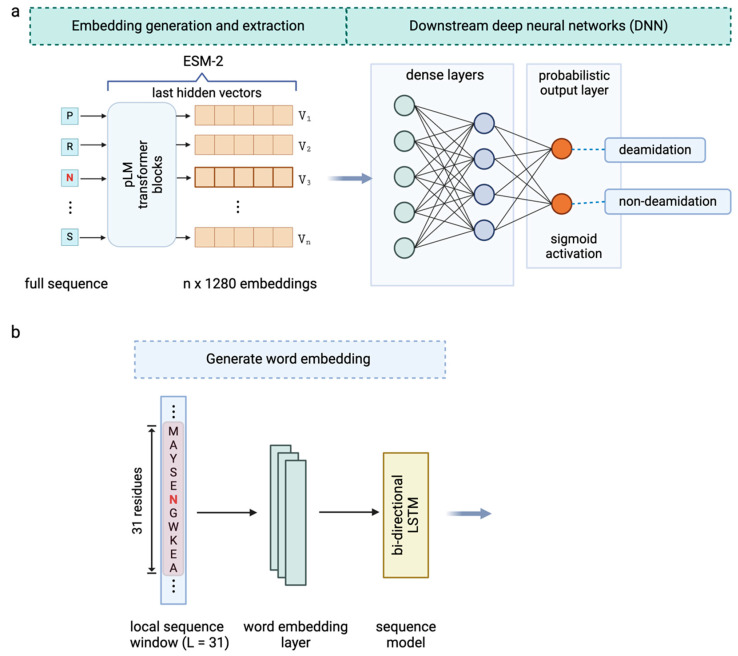
(**a**) The overall model architecture where ESM-2 embeddings corresponding to the potential deamidation sites were directly extracted and fed to downstream deep neural networks (DNNs) for the deamidation site classification. (**b**) Word embedding generation for local sequence windows of size 31 amino acids centered on the deamidation site of interest, followed by a bi-directional long short-term memory (LSTM) model to extract the local sequence information.

To leverage the pretrained ESM-2 model for encoding the sequence representations, the model takes the entire antibody sequence, including the sites of interest, as the input and returns the per residue representations of the full-length antibody. The outputs from the ESM-2 consist of residue-level sequence embeddings with dimensions of n×1280 (Figure 3a), where 1280 represents the dimension of the embeddings and n is the length of the amino acid sequence. These embedding features were then fed into downstream neural networks and trained to discriminate the antibody deamidation sites. This process, typically referred to as transfer learning [42], capitalizes on the knowledge gained from a previous task (in this case, the pretraining of the pLM) to improve the performance in new tasks (such as deamidation prediction) by reusing the learned feature representations, especially when the previous task was data-rich and the new tasks have limited labeled data. We have applied transfer learning by using a simple deep neural network (DNN) to fine-tune the downstream deamidation prediction task. The DNN comprises two hidden layers, each followed by a dropout layer to prevent overfitting. The overall model architecture, utilizing only ESM-2 embeddings as surrogate features for the deamidation site prediction, is depicted in Figure 3a. The detailed parameters associated with this architecture are provided in Table S2. Notably, concordant with previous findings [37,43,44], these pLM-derived features do not require sophisticated architectures to be adapted to new predictions.

The performance metrics of this model architecture are listed in Table 1. With an achieved accuracy of 94.4% and 0.798 and 0.728 for precision and recall, respectively, we showcase the possibility of predicting antibody deamidation sites using the ESM-2 protein language model taking only sequences as the input. Note that this is distinctly different from conventional sequence-based computational approaches, which simply convert selected sequence segments into static matrices; herein, the pLM derives context-dependent embeddings encompassing the intricate sequence–context relationships of the full-length antibody sequence. For each site of interest (the N or Q residue), the representation is transformed into a contextualized 1 × 1280 dimension vector, corresponding to 1280 meaningfully assimilated descriptors about this residue learned from the pretrained pLM. The effectiveness of complex unsupervised learned feature representations has also seen success in several other domain-specific tasks [43,45,46,47], outperforming handcrafted descriptors such as the one-hot encoding (OHE) of amino acids, k-mer motif counts, secondary structures, and backbone angles.

### 3.3. Enhanced Prediction by Combining ESM-2 Embedding and Local Sequence Information

Prior studies have highlighted the role of the local structural environment [25] and neighboring residues [48] in influencing the deamidation status of specific sites. Several local sequence motifs, such as NG, NS, and NN, among others, have been identified as correlating with the occurrence of antibody deamidations [18,49,50]. Recent investigations have demonstrated that these local sequence motifs, when integrated with additional structural or physicochemical property descriptors, can effectively predict deamidation hot spots [19,21,24,25]. Drawing from these insights, our study integrates the local sequence information with the global contextual information captured by the pretrained ESM-2 model. We trained a meta-classifier on the combined learned features. To transform the local sequences into numerical inputs understandable by the model, we constructed local sequence windows centered on the potential deamidation sites and then utilized supervised word embedding to capture the localized interactions among the amino acids surrounding the N/Q residues (Figure 3b). Subsequently, we employed bi-directional LSTM, a recurrent neural network (RNN) sequence model, to extract the features reflecting the associations and influences of neighboring amino acids within the defined sequence window. 

**Figure 5:**
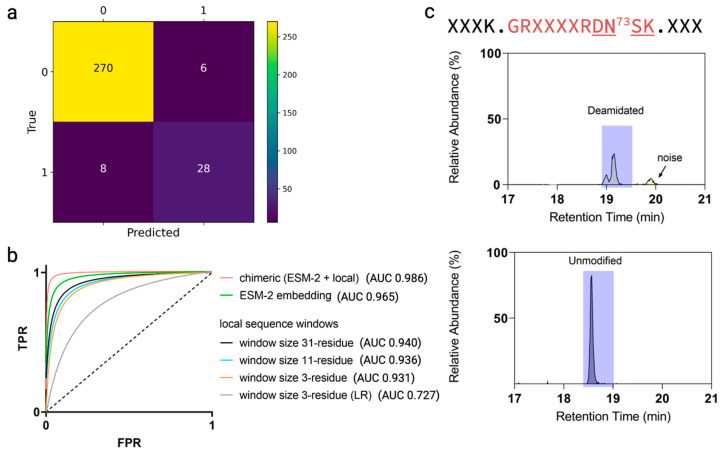
(**a**) Confusion matrix of the independent test dataset involving a total of 312 potential deamidation sites, with 276 experimentally confirmed inactive sites and 36 confirmed active sites. Specifically, within the confusion matrix, the model assigned 270 actual inactive sites as non-deamidated; the model identified 28 actual active sites as deamidation hot spots. Additionally, the model mistakenly assigned six inactive sites as deamidation hot spots (overprediction); and the model overlooked eight active sites and assigned them as inactive sites (underprediction). (**b**) The receiver operating characteristic (ROC) curves and area under the curves (AUCs) for the independent test set predictions with different models. The chimeric model outperformed the rest. (**c**) The extracted ion chromatograms corresponding to the deamidated and unmodified peptides via LysC digestion enabled experimental confirmation of the CDR N73 deamidation status.

Prior to incorporating the local sequence information into the ESM-2 embeddings, it was crucial to determine the optimal size of the sequence window. To achieve this, we processed various window sizes centered around the site of interest with an equal number of neighboring amino acids ranging from three to sixty-one. These sequences were forward-passed to the model through fivefold cross-validation using the deamidation training dataset; the MCC score was used as the metric for identifying the optimal window size. Of note, a window size of three corresponded to the 3-mer sequences incorporating the immediate adjacent residues (before and after) of the potential deamidation site as the input. In our study, a window size of three represented the minimal window sequence size. We interrogated the predictive capabilities of these local sequences solely using just the flanking sequences as the input to the base model depicted in Figure 3b without additional descriptors by gradually increasing the number of neighboring residues while maintaining an equal number of residues on both sides. The MCC values plotted against different window sizes are illustrated in Figure S4a. Briefly, the model performance saw a steady increase as the sequence window size was enlarged—a trend that was anticipated, because excessively short windows are likely to convey limited local sequence information. However, the MCC reached a plateau at approximately 31 amino acids. In Figure S4b–f, we also visualize the effect of word embedding and window sizes, in terms of the model’s ability to correctly distinguish a deamidation hot spot from an inactive set. With supervised word embedding, a window size of thirty-one amino acids (Figure S4e) outperformed a window size of three amino acids (Figure S4d); both performed significantly better than when supervised word embedding was not used (Figure S4c, 3-mer LR regression). Given all these, we selected a window size of 31 as optimal for the supervised word embedding. Window sizes beyond 61 residues were not explored owing to the computational burden associated with excessively long sequences. The detailed results for each window size are provided in Table S3. Noteworthily, we also tested local sequence models of different window sizes using an independent test set (Figure 5b); the outcome indicated that local sequences alone as predictors may not be as effective as using ESM-2-generated embeddings in terms of the deamidation prediction.

**Figure 4:**
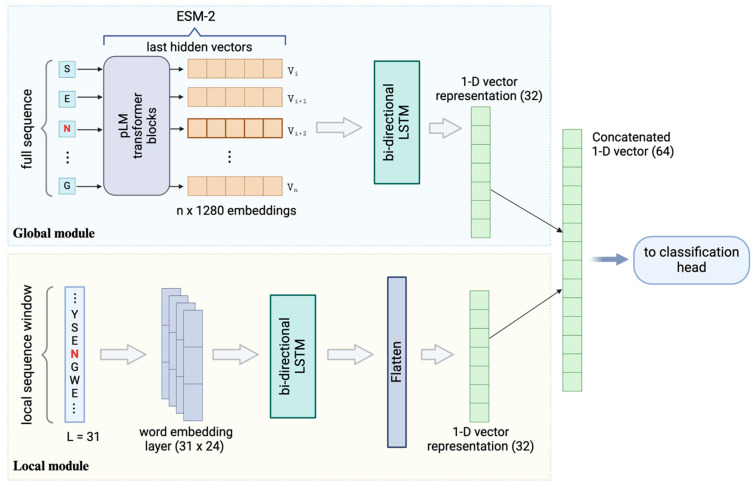
The overall architecture of the chimeric model for enhanced deamidation site classification. The architecture integrates the global module (ESM-2 embeddings) and local module (word embeddings) and combines the two output vectors using a concatenation layer followed by a simple DNN-based classification head. The detailed hyperparameters and layer settings are listed in the Supplemental Information.

The final architecture highlights a “chimeric” model comprising two processing modules (Figure 4), namely a local module that learns sequence information from the localized, windowed sequences and a global module that captures complex global contextual embeddings from the full-length protein sequences. Note that both modules directly take raw protein sequences as the input: there is no additional requirement for sequence alignment or handcrafted structural or physicochemical features. Each module independently encodes and processes the sequences, yielding 1-D vector as outputs. In order to integrate the learned features by the two modules, we concatenated the vectors from both sources and trained a fully connected (FC) neural network classification head as a meta-classifier (Figure 4 and Figure S5). The output of the classifier yields a probabilistic distribution ranging between 0 and 1, indicating the probability of being deamidated. This architecture was selected following the fivefold stratified cross-validation. Essentially, during this process, we ensured that each fold retained the same proportion of classes as the original dataset, thereby minimizing bias and improving the reliability of the model evaluations. We explored various other model architectures such as logistic regression, random forest, ANN, 1D-CNN, and RNN, alongside different hyperparameters including hidden layer numbers, neuron counts per layer, and optimizers. Moreover, we also implemented an early stopping mechanism to optimize the training and prevent overfitting. The hyperparameters used in the final architecture are listed in Table S4.

### 3.4. Performance Evaluation of Models

Given the imbalanced class distribution within the dataset, namely the prevalence of inactive N/Q deamidation sites vastly outnumbering deamidation hot spots (Figure 2b), the accuracy metric alone proved insufficient for assessing the classifier performance in this study. In extreme cases, where the model simply designates every site of interest as inactive, it may still achieve an 87.9% accuracy rate. Consequently, precision, recall, and specificity metrics were incorporated to provide a more comprehensive evaluation of the model performance. The metrics of precision and recall are particularly pertinent for an imbalanced dataset. Precision quantifies the classifier’s ability to accurately predict positive instances (i.e., the deamidation hot spot) relative to all predicted positive cases. Meanwhile, recall (also known as the true positive rate or TPR) assesses the classifier’s success in identifying deamidation hot spots among all the experimentally confirmed positives. In the context of deamidation site classification, striving for high precision and recall is essential for understanding the model’s capacity to distinguish deamidation hot spots amidst the predominant population of non-hot spots. 

The evaluation of the chimeric model involved performing fivefold stratified cross-validation on the training dataset and comparing it with the base models using either global contextual embedding or local sequence windows. The model performance was assessed using six metrics: accuracy, precision, recall, specificity, F1-score, and the Matthews correlation coefficient (MCC). In addition, receiver operating characteristic (ROC) curves were plotted for visualization purposes, with the area under the curve (AUC) calculated as an additional metric. Further elaboration on the performance metrics and their corresponding equations can be found in the Supplemental Information.

The comparative performance analysis (outlined in Table 1) revealed a notable distinction in the performance metrics. The mean MCC of the local sequence base model stood at 0.673 ± 0.030, while the ESM-2 base model exhibited an improved performance with a mean MCC of 0.731 ± 0.027. Remarkably, the chimeric meta-classifier exhibited a mean MCC of 0.787 ± 0.038, alongside enhanced performance metrics, including a mean accuracy of 0.956 ± 0.014, a mean precision of 0.836 ± 0.059, a mean recall of 0.789 ± 0.036, and a mean F1-score of 0.812 ± 0.031. 

To sum up, the chimeric model, which unites both global contextual embeddings and local sequence information from the two base models, outperforms any individual base model. Within the predominantly imbalanced training dataset containing 276 active deamidation sites (hot spots) and 2009 inactive sites, the chimeric model accurately identified 218 deamidation hot spots and 1966 inactive sites. Notably, approximately 84% of the predicted deamidation hot spots were corroborated as active sites in the peptide mapping experiments.

### 3.5. Independent Dataset Predicting Deamidation Hot Spots

To rigorously test the “chimeric” model performance for deamidation hot spot predictions, we used an independent dataset. The dataset composed of six antibodies, including five in-house antibodies and NISTmAb, all of which were subjected to automated peptide mapping following the identical handling and incubation at 40 °C at a pH of 8.0 for up to 8 weeks as described in Figure 1. Of the 312 total potential deamidation sites in this dataset involving N and Q residues, the chimeric model achieved an accuracy of 95%; 36 were labeled as true deamidation hot spots with the remaining 276 as inactive sites. The chimeric model correctly identified 28 deamidation hot spots with only six false positive cases among the deamidation inactive set; specifically, the model overpredicted six deamidation events that were not experimentally observed, while underpredicted eight cases (Figure 5a). Interestingly, one true positive deamidation event, revolving around the CDR N73 deamidation of antibody-2, captured by the chimeric model prediction was, however, overlooked in the peptide mapping to begin with, owing to the short peptide generated from tryptic digestion eluted with solvent front, causing the loss of sequence coverage, which included the asparagine site of interest. A follow-up LysC-based peptide mapping experiment confirmed this site as a true positive (Figure 5c). In Table S5, we show the prediction outcomes for NISTmAb, antibody-1, and antibody-2 from this dataset and highlight the deamidation hot spots. Additionally, we used the AUC value of the receiver operating characteristic (ROC) curve to benchmark the chimeric model’s performance and compared with other models such as the ESM-2 only (without the local module) model and several local sequence models using different window sizes (Figure 5b). As shown, the chimeric model demonstrated an AUC of 0.986, the highest among all the models evaluated.

Additionally, we also compared the performance of the chimeric model with other published deamidation classifiers, in order to assess the relative performance. We computed the performance metrics, including the accuracy, precision, recall, MCC, and specificity. It was imperative that the same training and test datasets were used for all the classifiers tested; herein, we applied to our independent dataset several different classifiers available from the literature. These included the structure-based decision tree model by Yan et al. [25] and a random forest model by Jia et al. [21]; we also included a sequence-based method called NGOME [22] by using the web server that directly took the sequence input with the default parameter settings [23]. Last but not least, we also compared all these approaches to a simple empirical method, which simply flagged all the NG, NS, and NN motifs to be deamidated. 

In Table 2, the comparison results among these different approaches are listed. As shown, the chimeric model achieved the highest MCC and accuracy. It did not achieve the highest precision performance, but our model still performed well in terms of the precision metric, only registering a lower value by 0.01 unit. Regarding the recall metric, the canonical NG/NS/NN motif-based approach gave an exceptionally high recall score of 0.944; however, these motifs are not always liable to deamidation, because these canonical motifs tend to overpredict deamidations, as evidenced by the significantly lower precision score of 0.586. 

### 3.6. Quantitative Deamidation Extents Prediction

**Figure 6:**
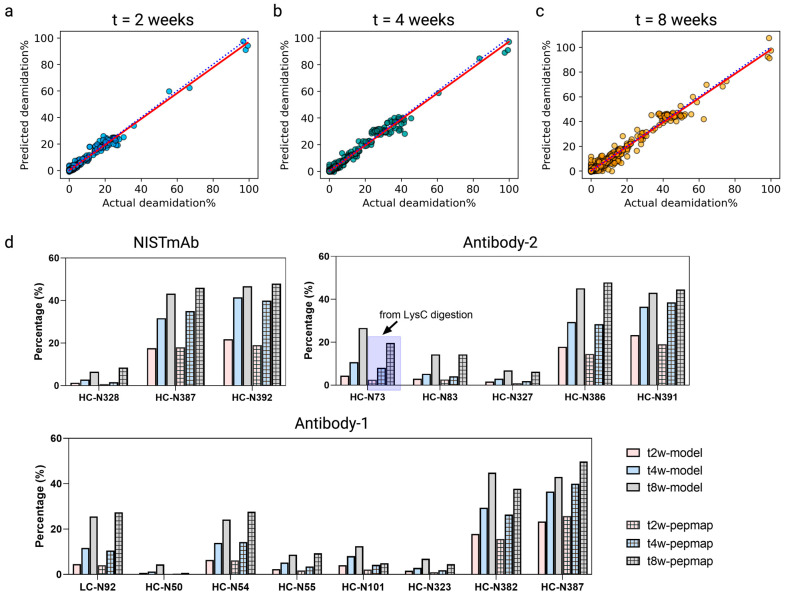
Chimeric model quantitative deamidation extent predictions evaluated by plotting the predicted deamidation values (y-axis) versus the peptide mapping determined values (x-axis) at 2-week (**a**), 4-week (**b**), and 8-week (**c**) time points. Linear regression curves (the red solid line) were generated and overlayed with diagonal lines (the blue dotted line) for visual evaluation purposes. (**d**) Bar graphs comparing the model-predicted deamidation extents and the corresponding experimentally determined deamidation extents for NISTmAb, antibody-1, and antibody-2 at time points t = 2, 4, and 8 weeks.

We show that the described architecture can go beyond classifying binary deamidation statuses to, further, quantitively predict the deamidation extents for future time points. This requires a simple adjustment of the model output layers by adding a regression head, which outputs three neurons corresponding to the three time points at t = 2, 4, and 8 weeks (Figure S5), followed by supervised learning using the experimentally measured deamidation extents at each time point as labels. We were able to train the model to provide quantitative deamidation information. In Figure 6a–c, we visualize the model performance based on fivefold cross-validation using the training dataset, where the predicted deamidation extents were plotted against the corresponding experimental deamidation percentages at different time points. At each time point, we performed linear regression as denoted by the solid red straight line, whereas the dotted lines represent the hypothetical 45° diagonal line where the measured deamidation levels are equal to those predicted. Overall, the regression model demonstrated good quantitative predictions for the deamidations. 

To further validate, we also tested using the same in-house independent test dataset that contained five in-house antibodies and NISTmAb. Shown in Fig 6d are the comparative results between the predicted deamidations for the hot spots and the actual peptide mapping measured deamidations for Antibody-1, Antibody-2, and NISTmAb, where the model accurately predicted the deamidation levels. For NISTmAb, the predicted deamidation extents for its designated three deamidation hot spots (N328, N387, and N392 of the heavy chain) aligned well with the measured levels; despite the comparable values among the prediction and experimental results, the model designated N328 as a hot spot, whereas, in reality, the true label for N328 is inactive (Table S5). In Antibody-1, the model’s quantitative predictions were in good agreement with the peptide mapping measurements for all time points with one exception of a marginally overpredicted deamidation on the heavy-chain N50. Although this site was labeled as inactive because it gave low deamidations experimentally throughout the 8-week time course (Figure 6d), the model classified it as a hot spot (Table S5); nevertheless, it is reassuring to see that the model’s regression only assigned low levels of deamidations. Most interestingly for Antibody-2 was the CDR deamidation of N73 on the heavy chain, while the model assigned this site as a deamidation hot spot and provided quite notable levels of deamidation predictions as shown in Table S5; experimentally, zero deamidation was detected in the peptide mapping in the first place owing to the sequence coverage loss by the small tryptic peptide (peptide DN^73^SK, Figure 5c) generated from trypsin digestion—the peptide was eluted with solvent front during the LC-MS; therefore, there was no coverage for N73 on the heavy chain. Fortunately, we were able to confirm the N73 deamidation status by conducting a LysC digestion peptide mapping, which rendered the peptide longer (namely, less hydrophilic with better retention) carrying the site of interest (Figure 5c and Figure 6d). By and large, this is a rare but interesting scenario highlighting that the model-based approach can overcome certain intrinsic limitations from the experimental approach. 

### 3.7. Model Implementation for High-Throughput Screening Drug Candidates

**Figure 7:**
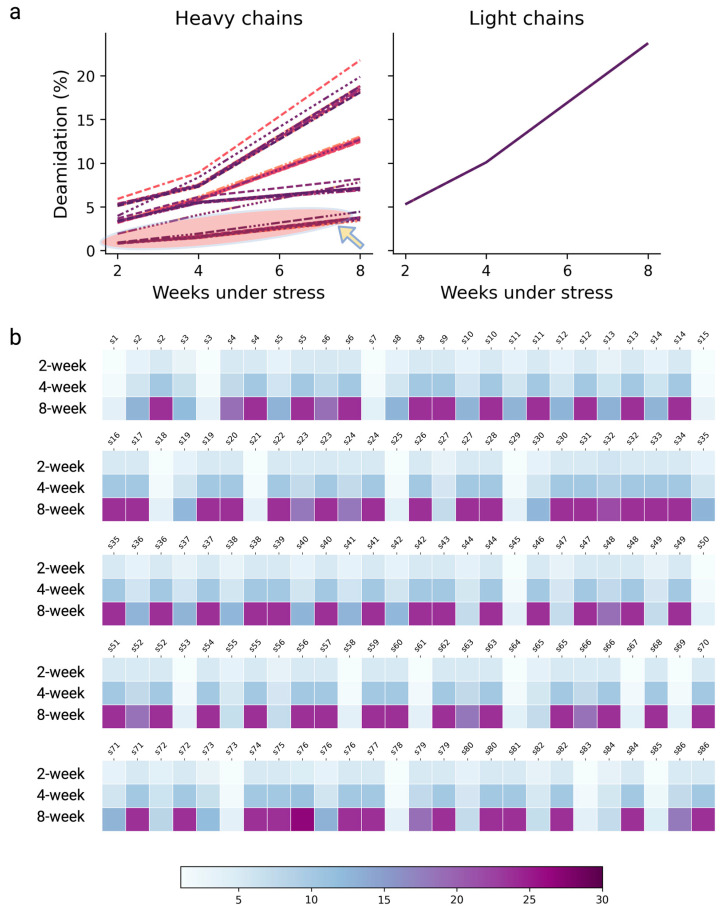
(**a**) A pilot study involving 86 different clones of antibodies, where the model projected the quantitative deamidation levels for each sample under the condition of a pH of 8.0 and 40 °C for 2 weeks, 4 weeks, and 8 weeks by taking the FASTA sequence files as the input. The circled region designates an identified panel of eight antibodies that exhibited a low deamidation liability on the heavy chain. (**b**) Heatmap compiling the residue-specific, time-dependent deamidation extents predicted by the model; each column refers to a specific N or Q residue (information masked) assigned as a deamidation hot spot of the designated clone.

In the drug discovery space, effective drug screening and triage play pivotal roles in identifying the optimal drug candidate, bringing about early de-risking, and accelerating the biologics design to development [51]. In addition to experimental screening approaches, computational screening and triaging have become a disruptive technique enabling the identification and optimization of drug candidates and advance lead selections [52,53]. In this work, we ran a pilot study involving 86 clones from different transfection pools and fed only the FASTA sequences to the model framework for the deamidation hot spots assignment and deamidation extents projection. All these clones shared a common light chain, but the heavy-chain sequences were vastly different. The model was able to project the deamidation extents and identified a panel of eight clones that potentially exhibited lower deamidation liabilities, as can be seen from Figure 7a where the heavy-chain sequences of these antibodies are predicted to carry less deamidation (<5%) even under 8 weeks of stressed conditions at a pH of 8.0 and 40 °C. The residue-specific deamidation profiles of each sample were further elucidated as a heatmap (Figure 7b).

Notably, this screening process only took several minutes; in contrast, we estimate it may take up to 4 months to harvest comparable information experimentally for the 86 clones, given the lengthy processes including the samples’ forced degradation treatment, peptide mapping sample preparation, LC-MS/MS data acquisition, and data processing. In fact, one may find that it is difficult to justify experimentally measuring all these samples in the discovery phase considering the potential time and resources required to invest upfront. Nevertheless, we demonstrate this model-based approach as a potential high-throughput screening and triage tool that facilitates the access of deamidation liability profiling, because this information not only reduces experimental burdens but also, when in conjunction with other experimental efforts, can potentially ensure more effective drug lead selection and optimization. 

## 4. Discussion and Conclusions

In this work, we showcase that it is possible to accurately predict antibody deamidations by using the state-of-the-art protein language model (pLM) framework. We described a systematic workflow capacitated by a novel high-throughput peptide mapping procedure, followed by pLM-driven deep learning. The pLM used here was ESM-2. Our primary objective was to highlight the potential of using protein language models to extract latent, context-dependent information feasible for assimilated protein features and to implement automated peptide mapping to generate a large amount of high-quality residue-specific data to fulfill the need for task-specific, supervised machine learning. The automation platform outlined here is an elaboration of a previously described workflow [54], with the added functionality of automated sample concentration normalization. 

Compared to conventional machine learning methodologies in the context of deamidation predictions that require various handcrafted descriptors from structural and/or physicochemical aspects in addition to protein sequences as the input, the pLM-based methodology greatly simplifies the input and only requires the primary sequences. For optimal performance, we investigated several different model architectures and settled on a chimeric design that incorporates two base models working cohesively, extracting global sequence representations and local sequence information, respectively. The novelty of this approach is to use a pretrained protein language model (ESM-2) harvesting the global contextual embeddings for the sites in conjunction with the use of supervised word embedding mining the local sequence dependencies. We demonstrated that the chimeric model performed well in both deamidation classification and regression tasks. Additionally, these findings may suggest that the information on the evolutionary context of a sequence, more specifically, the potential rules pertinent to deamidation occurrences, is already embedded in the large language model ESM-2.

The improved performance of this model is most likely owing to the adoption of contextual protein language models that extract features from the overall protein sequences for the site of interest. These latent features have shown great flexibility and robustness in domain-specific tasks, even with sparse datasets where transfer learning, which entails training models on large datasets to study scarce datasets, becomes very useful [55]. In this work, despite high-throughput automation, the available deamidation instances are still relatively few, and the overall dataset is imbalanced. This, however, makes a good use case for combining language model-based approaches in conjunction with transfer learning. Specifically, the embeddings learned from the pretrained pLM (i.e., ESM-2) are essentially distilled knowledge obtained through the data-rich pretraining objectives; this knowledge was then used to improve the downstream deamidation prediction tasks by feeding it to the existing deamidation dataset supervised by the peptide mapping-determined readouts. To the best of our knowledge, this work is the first using the distilled knowledge gathered from large pretrained pLMs for the prediction of deamidations; of note, pLMs have been used for other post-translational modification predictions, such as succinylation [37] and phosphorylation [36]. Our data suggest that the pLM-derived representations are versatile, adaptive features; the analyses of the pLM representations have indicated that pLMs intrinsically learn essential biologically relevant features [29]—a likely explanation why a simple model architecture is sufficient to achieve competitive performances.

Despite the exceptional performance, an inevitable limitation of the outlined chimeric approach is the lack of clear insights as to what specific features are crucial for the learning and how they contribute to determining deamidations. This limitation echoes with the inherent lack of interpretability of protein language models or any large language models (LLMs); currently, a comprehensive understanding of the inner workings of large language models remains elusive. In contrast, take the local sequence base model as an example, although it was less predictive compared to the chimeric model or to the ESM-2 base model (Table 1): it is advantageous in that it is easily interpretable and provides insights regarding the top sequence motifs learned that are most predictive of deamidations. For instance, with the supervised word embedding using a window size of three amino acids, the local sequence model found that the top three deamidation X+1 motifs are NG, NS, and NN, in good agreement with previous findings that the canonical motifs NG, NS, and NN are among the most common in deamidation degradation [8,17,18,56] and that glycine and serine are critical residues affecting deamidation owing to their steric and catalytic effects [56]. Interestingly, this local sequence model also identified sequence motifs such as SN, EN, and WN as the top three X-1 motifs. Chelius et al. have, concordantly, reported the highest level of deamidation in terms of X-1 motifs, including SN, EN, and LN [50].

Our model can serve as a surrogate for labor-intensive experiments, particularly during the preclinical phases when managing substantial numbers of samples with diverse sequences and complexities. This approach significantly conserves time and resources. Additionally, following the screening campaign, the model can be deployed to assess the lead antibody candidates, providing insights into the potential critical quality attributes (pCQAs) that can guide drug developments and process optimizations. Notably, this presented workflow is not limited to antibody deamidations but, with minimal adjustment, is extendable to other sequence liabilities, such as Asp isomerization, Met and Trp oxidation, Tyr sulfation, etc. In particular, Asp isomerization liability has been reported on antibodies impacting their stability and potency [48,57]. Noteworthily, we also observed sporadic isomerization modifications in our dataset under the condition of a pH of 8.0 and 40 °C; however, Asp isomerization has a higher rate at a lower pH (<5.5) [48,58]. While extending the protein language model-driven framework outlined here to Asp isomerization should be straightforward, challenges lie in the mass spectrometric detection and curation of a quality dataset as, unlike deamidations that render a +0.98 Da mass shift, isomerization has no net molecular mass change; instead, the detection and quantification of isomerization species largely depend on the chromatographic separation between isoAsp and Asp species. The use of ETD, rather than CID or HCD, for tandem mass fragmentations may assist in distinguishing isoAsp and Asp species and resolve residue-specific isomerization [59].

Considerations to further improve the performance of pLM-focused approaches may involve the following aspects: (i) conducting the supervised fine-tuning (FT) [60] or parameter-efficient fine-tuning (PEFT) [61] of the pretrained pLM to tailor the language model to more efficient transfer learning, including adapting to downstream tasks; (ii) combining pLM embeddings with additional descriptors, such as structural or physicochemical features; and (iii) as with any machine learning model, increasing the dataset size to help with accurate results.

## Figures and Tables

**Table 1:** Performance metrics for models harnessing different sequence representations in the prediction of deamidation using fivefold stratified cross validation on the training dataset.

Descriptors	Accuracy	Precision	Recall	Specificity	F1-Score	MCC
Local sequence only	0.932 ± 0.014	0.745 ± 0.044	0.679 ± 0.039	0.967 ± 0.019	0.710 ± 0.035	0.673 ± 0.030
Global embeddings only	0.944 ± 0.012	0.798 ± 0.049	0.728 ± 0.043	0.975 ± 0.016	0.761 ± 0.046	0.731 ± 0.027
Local + global embeddings	0.956 ± 0.014	0.835 ± 0.059	0.790 ± 0.036	0.979 ± 0.016	0.812 ± 0.031	0.787 ± 0.038

**Table 2:** Comparison of prediction performance using an independent test set. Values are rounded to three decimal places; highest value in each performance metric is highlighted in bold.

Classifier	Accuracy	Precision	Recall	MCC	Specificity
Decision tree model [25]	0.949	**0.833**	0.694	0.733	**0.982**
Random forest model [21]	0.952	0.818	0.750	0.757	0.978
NGOME [23]	0.942	0.781	0.694	0.705	0.975
NG, NS, and NN motifs	0.917	0.586	**0.944**	0.704	0.913
Chimeric model	**0.955**	0.823	0.778	**0.775**	0.978

## Data Availability

The model construct and layer hyperparameters are available in the Supplemental Information.

## Supplementary Materials

The following supporting information can be downloaded at https://www.mdpi.com/article/10.3390/antib13030074/s1, File S1: Supplemental Experimental and Methods; Figure S1: Overlay of ultraviolet (UV) chromatograms from NISTmAb samples located at diagonal well positions on a 96-well plate (A1-H8); Figure S2: The bufferfly plot of UV chromatograms of NISTmAb samples; Figure S3: Quantitative overview of deamidation instances (n = 2285) by their respective deamidation labels (hot spot versus inactive); Figure S4: Window size selection and histograms of predicted deamidation probability distribution by various models; Figure S5: Classification head and regression head; Table S1: Post-translational modifications (PTMs) quantitation results from replicate NISTmAb samples located at diagonal well positions on a 96-well plate (A1-H8); Table S2: Hyperparameters of the ESM-2-based DNN model; Table S3: Window size selection model performance details; Table S4: Hyperparameters of the chimeric model incorporating the global module and local module; Table S5: Independent test set prediction results taking NISTmAb, antibody-1, antibody-2 as examples.

## Abbreviations

Ab	Antibody
ANN	Artificial neural network
Asn	Asparagine
Asp	Aspartic acid
AUC	Area under the curve
CDR	Complementarity-determining region
CID	Collision-induced dissociation
cIEF	Capillary isoelectric focusing
CNN	Convolutional neural network
Cys	Cysteine
DNN	Deep neural network
DTT	Dithiothreitol
EDTA	Ethylenediaminetetraacetic acid
ESM	Evolutionary scale modeling
ETD	Electron-transfer dissociation
FN	False negative
FP	False positive
FPR	False positive rate
FTE	Full-time employee
GdnHCl	Guanidine hydrochloride
HCD	Higher energy collision dissociation
HTP	High-throughput
IAM	Iodoacetamide
LC-MS	Liquid chromatography–mass spectrometry
LSTM	Long short-term memory
Lys	Lysine
LysC	Endoproteinase Lys-C enzyme
mAb	Monoclonal antibody
MCC	Matthews correlation coefficient
Met	Methionine
MS/MS	Tandem mass spectrometry
pCQA	Potential critical quality attributes
pLM	Protein language model
PTM	Post-translational modification
QSAR	Quantitative structure–activity relationship
ReLU	Rectified linear unit
RNN	Recurrent neural network
ROC	Receiver operating characteristics
SASA	Solvent-accessible surface area
SD	Standard deviation
TFA	Trifluoroacetic acid
TN	True negative
TP	True positive
TPR	True positive rate
Trp	Tryptophan
Tyr	Tyrosine
UV	Ultraviolet
XIC	Extracted ion chromatogram
