# Supplementary material for: The Accurate Prediction of Antibody Deamidations by Combining High-Throughput Automated Peptide Mapping and Protein Language Model-Based Deep Learning
Source: Antibodies (Basel). 2024 Sep 10;13(3):74. doi: 10.3390/antib13030074 (PMC11417914; doi:10.3390/antib13030074)
Supplement: Supplementary file 1 [file antibodies-13-00074-s001.zip › antibodies-3171150-supplementary.pdf]

Supplemental Information

The Accurate Prediction of Antibody Deamidations by Combining High-Throughput Automated Peptide Mapping and Protein Language Model-Based Deep Learning

Ben Niu [1,*], Benjamin Lee [1], Lili Wang [2], Wen Chen [1] and Jeffrey Johnson [1]

[1] Discovery Biotherapeutics, Bristol Myers Squibb, San Diego, CA 92121, USA; benjamin.lee@bms.com (B.L.); wen.chen@bms.com (W.C.); jeffrey.johnson@bms.com (J.J.)
[2] Department of Molecular and Cell Biology, University of California, Berkeley, CA 94720, USA; lili.wang@berkeley.edu
* Correspondence: benniu720@gmail.com

List of Supplemental Information

File S1:
Supplemental Experimental and Methods:

- Automated peptide mapping
- LC-MS/MS data analysis
- Model performance evaluation

Figure S1: Overlay of ultraviolet (UV) chromatograms from NISTmAb samples located at diagonal well positions on a 96-well plate (A1-H8).

Figure S2: The bufferfly plot of UV chromatograms of NISTmAb samples.

Figure S3: Quantitative overview of deamidation instances (n = 2285) by their respective deamidation labels (hot spot versus inactive).

Figure S4: Window size selection and histograms of predicted deamidation probability distribution by various models.

Figure S5: Classification head and regression head.

Table S1: Post-translational modifications (PTMs) quantitation results from replicate NISTmAb samples located at diagonal well positions on a 96-well plate (A1-H8).

Table S2: Hyperparameters of the ESM-2-based DNN model

Table S3: Window size selection model performance details.

Table S4: Hyperparameters of the chimeric model incorporating the global module and local module.

Table S5: Independent test set prediction results taking NISTmAb, antibody-1, antibody-2 as examples.

File S1:
Supplemental Experimental and Methods

Automated peptide mapping

The fully automated peptide mapping sample preparation protocol was performed using a Lynx LM1200 system (Dynamic Devices). The robot features a pipetting arm with an individually addressable 96-channel pipetting arm, each channel having a maximum capacity of 1250 µL, alongside a plate gripper facilitating all plates movements during the procedure. The liquid handler deck was equipped with a BioShake Q1 (Q Instruments) to enable heating, cooling, and shaking required in the protocol.

To start, all samples from the source 96-well plate were transferred to the denaturing plate, where the concentrations of samples were normalized to a final concentration of 2.0 mg/mL. An aliquot of 30 µL denaturing buffer, a pre-made solution containing 7.9 M guanidine hydrochloride, 40 mM DTT, and 0.5 mM EDTA with 100 mM Tris at pH 7.2, was added to each sample. Next, the denaturing plate was transferred to the BioShake Q1 for incubation at 37°C with shaking for 30 minutes. After completion, the plate was retrieved and placed back to its original position. Following this, the plate gripper was used to remove the light-protective lid on the IAM plate; an aliquot of 25 µL of 100 mM IAM solution was added to each denatured sample. The incubation for IAM alkylation was conducted at room temperature for 30 minutes, with the light-protective lid placed on the sample plate. During this incubation, an aliquot of 1.8 mL dialysis buffer (containing 2 M urea, 150 mM Tris at pH 7.4) was transferred into each well of the microdialysis cartridge (Thermo Fisher Scientific). Upon completion of IAM alkylation, the light-protective lid was removed, aliquot of 60 µL of sample solution was then transferred to the microdialysis cassette through the portal on top. Owing to the small size of each portal, to ensure successful liquid transfer during this step, the pipette tips were guided to move down 1.0 mm into the dialysis cassette portal; this depth helped maintain a good seal between the pipette tips and cassettes. After this, the entire microdialysis cartridge was moved to the BioShake Q1 for a 90-minute dialysis period at 500 rpm. Following the microdialysis, the full volume of sample was aspirated from the dialysis cassettes and transferred to the trypsin plate where an aliquot of 20 µL 0.125 µg/µL trypsin solution was added to each sample. The solution was thoroughly mixed by pipetting arm, followed by moving the trypsin plate (with lid on) to the BioShake Q1 using the gripper for a 3.5-hour incubation at 37°C. The reaction was quenched by adding 20 µL 2.5% TFA solution to each sample upon completion of trypsin digestion. Finally, the trypsin plate with quenched digests, covered with lid, was plated onto the BioShake Q1 (end temperature set to 4°C). The final sample volume was ~100 µL, at concentration approximately 0.3 mg/mL.

LC-MS/MS data analysis

Data base searching of raw peptide mapping data files (in *.raw data format) were batch-processed using Byos software against a custom-built database containing the antibody sequences of interest. Decoy sequences were included during the search. The searching parameters were set to consider only fully tryptic cleavage peptides (cleavages at C-terminal of Arg and Lys residues) with a maximum of 2 missed cleavage allowed. Mass tolerance window for precursor ions and product ions was 10 ppm, and 50 ppm, respectively. Maximum precursor mass was set to 15,000 Da. In terms of post-translational modifications (PTMs) settings, the alkylation of Cys-containing peptides by IAM, which adds a carbamidomethyl group (+57.0214 Da), was deemed as a fixed modification in

the search; whereas deamidation modification which renders a 0.9840 Da mass shift, was included as a variable modification. Other PTMs also included in the search are oxidation (+15.9949 Da), N-succinimide formation (-17.0265 Da), D-succinimide formation (-18.0106 Da), kynurenine formation (+3.9939 Da), pyroglutamate (-17.0265 Da), amidated proline (-58.0055 Da), C-terminal Lys (+128.0950 Da). Glycopeptide identification was performed simultaneously by incorporating the N-glycan 53 common biantennary glycan database. The final peptide identifications were filtered based on two criteria: (1) precursor ion intensity > 5e5 and (2) search score > 40. Careful inspection of MS/MS spectra for identified peptides and validation of PTMs quantification were conducted within Byos interface, in order to obtain confident residue-specific PTM assignment and quantification. For each individual, site-specific PTM, the quantification percentage was calculated based on the ratio of summed extracted-ion chromatograms (XICs) of all detected charge states, with respect to the modified and unmodified peptides, as described by the equation below:

$$PTM\% = \frac{\Sigma A_{modified}}{\Sigma A_{unmodified} + \Sigma A_{modified}} \times 100\%$$

Model performance evaluation

The predictive performance was evaluated by six metrics, namely, accuracy, precision, recall, specificity, F1-score, and Matthew's correlation coefficient (MCC); these metrics can be calculated from a standard confusion matrix comprising four components: True Positive (TP), referring to the count of actual deamidation sites predicted as active; True Negative (TN), referring to the count of actual non-deamidated sites predicted as inactive; False Positive (FP), referring to the count of actual non-deamidated sites predicted as active; and False Negative (FN), referring to the count of actual deamidation sites predicted as inactive. The calculations were based on the equations listed as follows:

$$accuracy = \frac{TP + TN}{TP + TN + FP + FN}$$

$$precision = \frac{TP}{TP + FP}$$

$$recall = \frac{TP}{TP + FN}$$

$$specificity = \frac{TN}{TN + FP}$$

$$F1\ score = \frac{precision \times recall}{precison + recall}$$

$$MCC = \frac{(TP \times TN) - (FP \times FN)}{\sqrt{(TP + FP) \times (TP + FN) \times (TN + FP) \times (TN + FN)}}$$

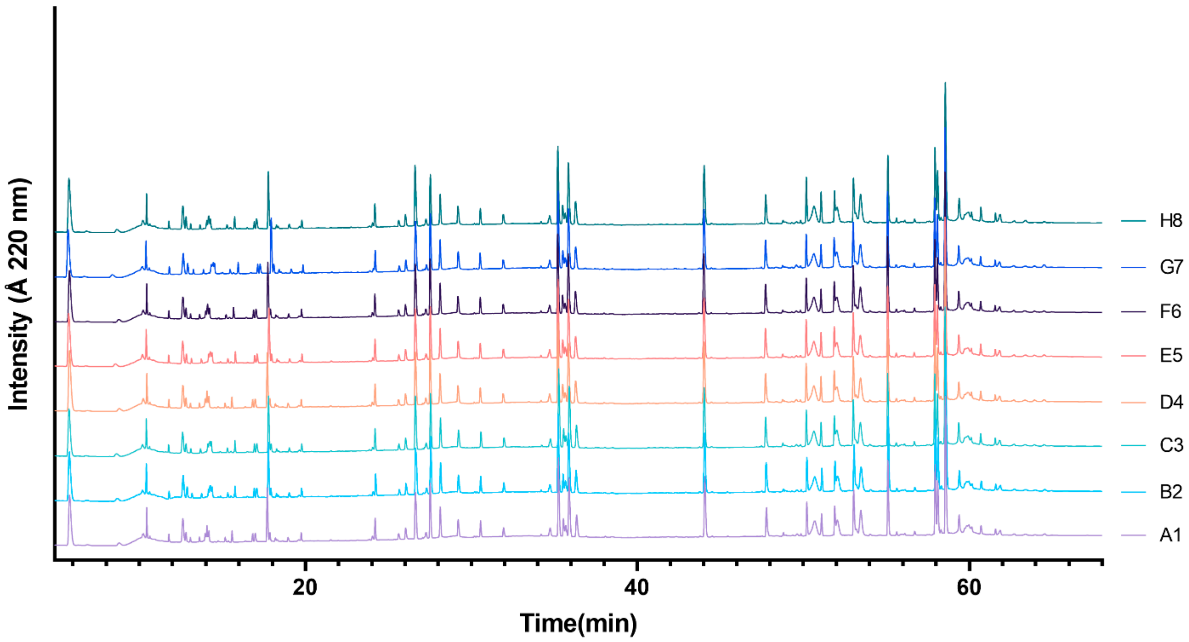

Figure S1. Overlay of ultraviolet (UV) chromatograms from NISTmAb samples located at diagonal well positions on a 96-well plate (A1-H8).

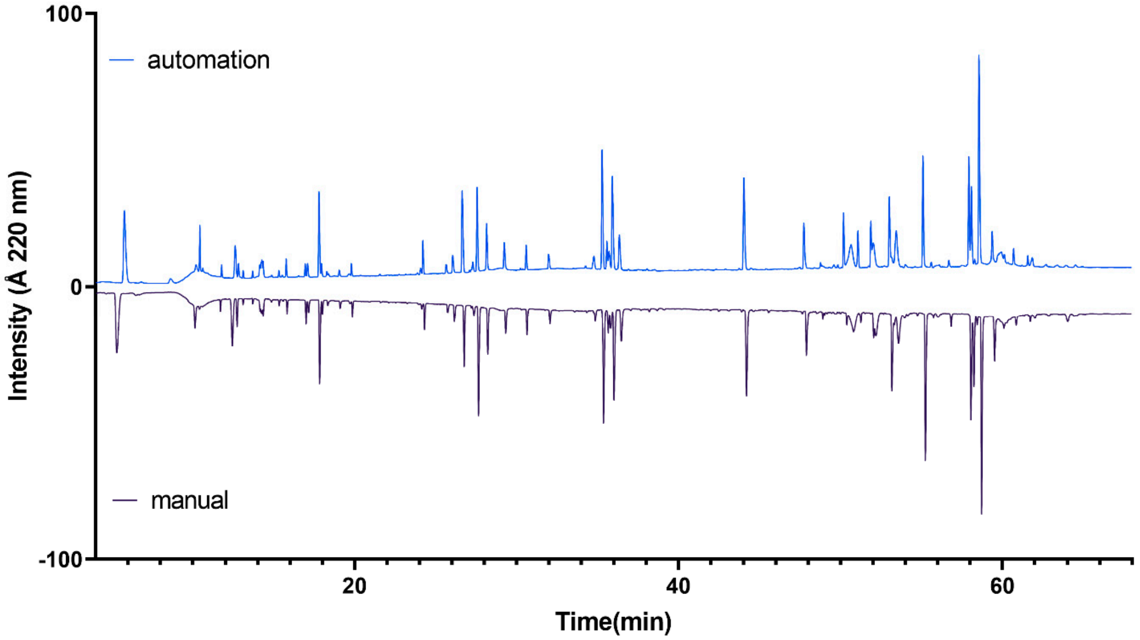

Figure S2. The bufferfly plot of UV chromatograms of NISTmAb samples, comparing the automated tryptic peptide mapping protocol (top) and manual protocol (bottom) showed that the digestion profiles between the two approaches were highly comparable.

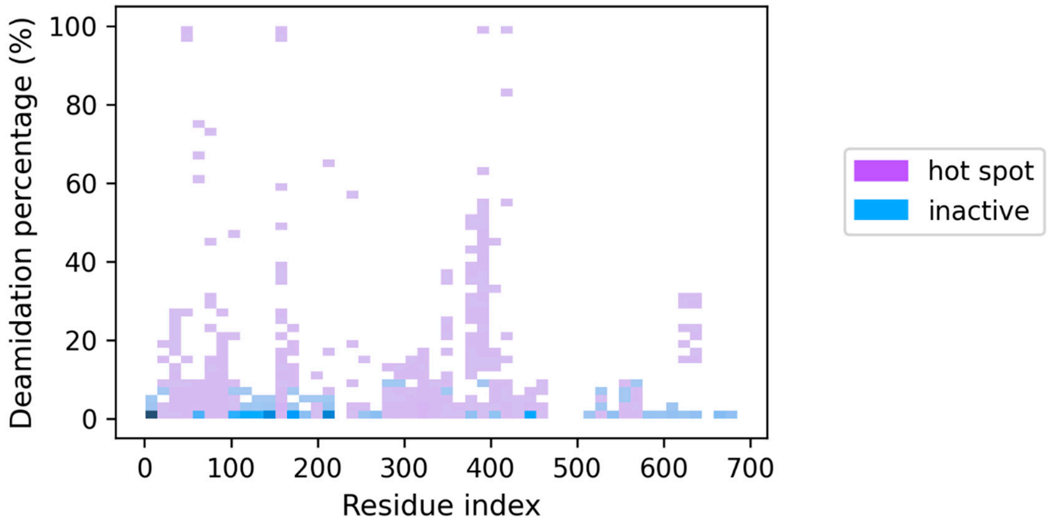

Figure S3. Quantitative overview of deamidation instances (n = 2285) by their respective deamidation labels (hot spot versus inactive). Each deamidation instance was accompanied with experimental measurement of deamidation levels (y-axis) at t=2week, t=4week, and t=8week three time points; the x-axis refers to the corresponding residue index for site of interest. The overall distribution of measured deamidations indicate that residues flagged as inactive exhibited lower deamidation extents, whereas residues flagged as hot spot showed much higher extents.

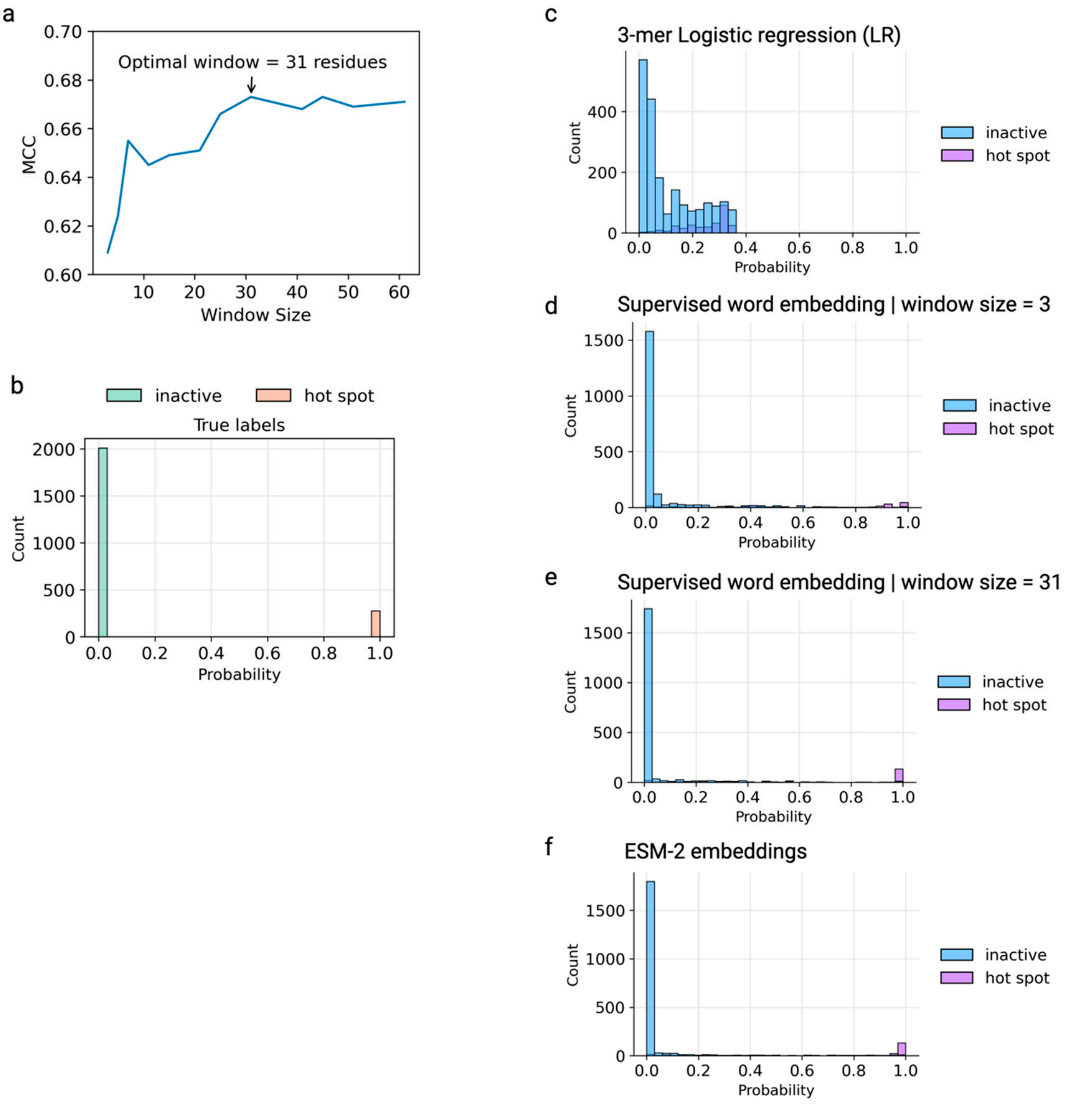

Figure S4. Window size selection and histograms of predicted deamidation probability distribution by various models. a) The different window sizes plotted against the corresponding MCC values, which gradually increased and reached plateau around window size of 31 amino acids. b) Histogram of training dataset (n = 2285) showing the distribution of deamidation labels along the probability scale, with deamidation hot spot at 1 and inactive set at 0. c-f) Histogram of fivefold cross-validation using training dataset (n = 2285) showing the distribution of predicted deamidation probabilities colored by true deamidation labels (inactive vs. hot spot), predictions were from 3-mer logistic regression without using supervised word embedding (c); supervised word embedding with window size of 3 amino acids followed by LSTM model (d); supervised word embedding with window size of 31 amino acids followed by LSTM model (e); the embeddings from pretrained ESM-2 followed by DNN model (f).

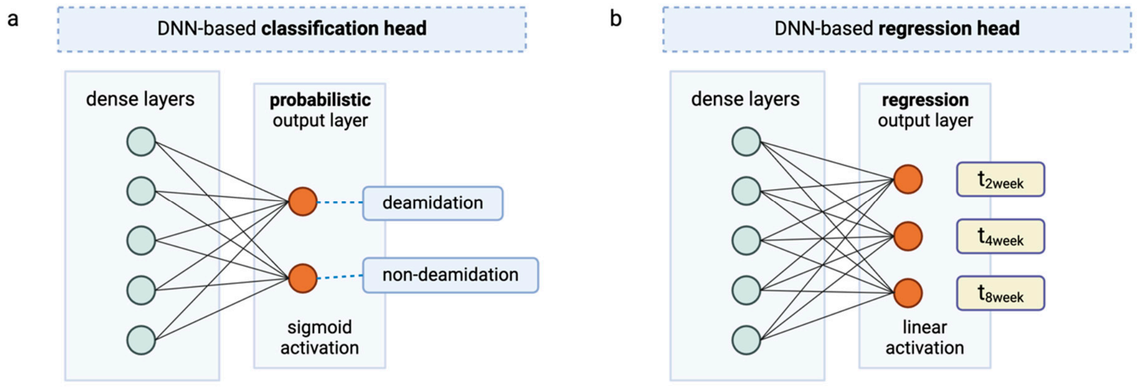

Figure S5. Classification head and regression head. The architecture of deep neural network (DNN)-based classification head (a) and regression head (b) used in the chimeric model. Detailed parameters settings refer to Table S4.

Table S1. Post-translational modifications (PTMs) quantitation results from replicate NISTmAb samples located at diagonal well positions on a 96-well plate (A1-H8).

Chain	Peptide Sequence	Residue	Modification	Sample replicate # (plate well location)								Mean±SD
				A1	B2	C3	D4	E5	F6	G7	H8	
Heavy chain	QVTLR	Q1	N-term pyroE	100	100	100	100	100	100	100	100	100±0
	VTNMDPADTATYYCAR	N86	Deamidation	0.2	0.1	0.1	0.2	0.2	0.2	0.1	0.1	0.2±0.1
	DTLMISR	M255	Oxidation	3.0	3.2	3.1	3.1	3.3	3.2	3.0	3.2	3.1±0.1
	TKPREEQYNSTYR	N300	G0F-GN	3.5	3.5	3.8	3.7	3.4	3.6	3.8	3.6	3.6±0.1
			G0F	39.0	38.8	38.4	39.4	38.5	37.9	38.2	37.8	38.5±0.5
			G1F	39.0	39.6	38.8	39.4	39.7	39.5	40.1	39.2	39.4±0.4
			G2F	5.3	5.2	5.3	5.3	5.5	5.1	5.3	5.0	5.3±0.2
	GFYPSDIAVEWESNGQPENNYK	N387	Deamidation	0.9	1.1	1.0	1.0	1.1	0.9	0.9	1.1	1.0±0.1
		N392	Deamidation	1.2	1.1	1.1	1.2	1.3	1.0	1.1	1.1	1.1±0.1
	WQQGNVFSCSVMHEALHNHYTQK	M431	Oxidation	0.9	0.8	0.8	1.0	0.8	0.9	0.9	0.8	0.9±0.1
	SLSLSPG	C-term	C-term Lys	3.2	3.4	3.1	3.2	3.2	3.4	3.1	3.3	3.2±0.1
Light chain	SGTASVVCLLNNFYPR	N136	Deamidation	0.2	0.1	0.1	0.2	0.2	0.1	0.1	0.1	0.1±0.1
	VYACEVTHQGLSSPVTK	Q198	Deamidation	0.0	0.1	0.1	0.1	0.1	0.0	0.1	0.0	0.1±0.1

Table S2. Hyperparameters of the ESM-2-based DNN model

**Parameters**	**Setting / Value**
Input_shape	(1280,)
Dense layer_1 # of neurons	128
Dense layer_1 activation function	ReLU
Dropout rate	0.5
Dense layer_2 # of neurons	32
Dense layer_2 activation function	ReLU
Dropout rate	0.5
Final layer # of neurons	1
Final layer activation function	Sigmoid
Epochs	150
Optimizer	RMSprop
Learning rate	1e-4
Callback	EarlyStopping

Table S3. Window size selection model performance details. Window size of 31 amino acids was selected using MCC as metric. In addition, other metrics including accuracy, precision, recall, specificity were listed.

Window size	MCC	Accuracy	Precision	Recall	Specificity
61	0.671	0.932	0.745	0.676	0.968
51	0.669	0.931	0.744	0.673	0.968
45	0.673	0.932	0.749	0.675	0.968
41	0.668	0.931	0.752	0.664	0.969
35	0.671	0.931	0.754	0.667	0.969
**31**	**0.673**	0.932	0.745	0.679	0.967
25	0.666	0.931	0.729	0.681	0.965
21	0.652	0.926	0.718	0.670	0.963
15	0.649	0.926	0.724	0.658	0.965
11	0.646	0.925	0.714	0.664	0.962
7	0.655	0.928	0.716	0.675	0.963
5	0.624	0.920	0.693	0.647	0.959
3	0.609	0.918	0.681	0.630	0.954

Table S4. Hyperparameters of the chimeric model incorporating the global module and local module.

Global module (ESM-2-based LSTM model)	
**Parameters**	**Setting / Value**
Input_shape	(1, 1280)
LSTM layer # of neurons	32
Dropout rate	0.3
Dense layer_1 # of neurons	32
Dense layer_1 activation function	ReLU
Local module (Supervised word embedding layer followed by LSTM model)	
**Parameters**	**Setting / Value**
Input_shape	(31,)
Embedding input dimension	23
Embedding output dimension	24
LSTM layer # of neurons	32
Dropout rate	0.4
Flatten layer	True
Dense layer_1 # of neurons	32
Dense layer_1 activation function	ReLU
Concatenation	
**Parameters**	**Setting / Value**
Concatenate layer shape	32 + 32
DNN-based classification head	
**Parameters**	**Setting / Value**
Dense layer_1 # of neurons	64
Dense layer_1 activation function	ReLU
Dropout rate	0.3
Final layer # of neurons	1
Final layer activation function	Sigmoid
Epochs	150
Optimizer	Adam
Learning rate	1e-3
Callback	EarlyStopping
DNN-based regression head	
**Parameters**	**Setting / Value**
Dense layer_1 # of neurons	256
Dense layer_1 activation function	ReLU
Dropout rate	0.3
Final layer # of neurons	3
Final layer activation function	Linear
Epochs	150
Optimizer	Adam
Learning rate	1e-3

Table S5. Independent test set prediction results taking NISTmAb, antibody-1, antibody-2 as examples. Only the residues predicted to be active deamidation (hot spot) are shown. The column of "Residue" denotes the site-specific deamidation; the column of "True Label" refers to the peptide mapping experimentally confirmed deamidation label (T for active, F for inactive). In the "Prediction" columns, the "Deamidation status" column refers to the model predicted deamidation status; followed by quantitative deamidation level predictions at three future time points (t= 2, 4, 8 weeks).

**Residue**	**True Label**	**Prediction**			
		Deamidation status	t_{2week}	t_{4week}	t_{8week}
Antibody-1					
Q3 ǀ L	F	F			
Q6 ǀ L	F	F			
Q27 ǀ L	F	F			
Q37 ǀ L	F	F			
Q38 ǀ L	F	F			
Q79 ǀ L	F	F			
Q89 ǀ L	F	F			
Q90 ǀ L	F	F			
N92 ǀ L	T	T	4.6%	11.7%	25.5%
Q124 ǀ L	F	F			
N137 ǀ L	F	F			
N138 ǀ L	F	F			
Q147 ǀ L	F	F			
N152 ǀ L	F	F			
Q155 ǀ L	F	F			
N158 ǀ L	F	F			
Q160 ǀ L	F	F			
Q199 ǀ L	F	F			
N210 ǀ L	F	F			
Q3 ǀ H	F	F			
Q6 ǀ H	F	F			
Q39 ǀ H	F	F			
N50 ǀ H	F	T	0.7%	1.3%	4.4%
N52 ǀ H	F	F			
N54 ǀ H	T	T	6.4%	13.9%	24.2%
N55 ǀ H	T	T	2.4%	5.2%	8.7%
N61 ǀ H	F	F			
Q62 ǀ H	F	F			
N101 ǀ H	T	T	4.1%	8.1%	12.4%
Q107 ǀ H	F	F			
N157 ǀ H	F	F			
Q173 ǀ H	F	F			
Q194 ǀ H	F	F			
N199 ǀ H	F	F			
N201 ǀ H	F	F			
N206 ǀ H	F	F			
N274 ǀ H	F	F			

<table>
<tr><td>N284 | H</td><td>F</td><td>F</td><td></td><td></td><td></td></tr>
<tr><td>Q293 | H</td><td>F</td><td>F</td><td></td><td></td><td></td></tr>
<tr><td>N295 | H</td><td>F</td><td>F</td><td></td><td></td><td></td></tr>
<tr><td>Q309 | H</td><td>F</td><td>F</td><td></td><td></td><td></td></tr>
<tr><td>N313 | H</td><td>F</td><td>F</td><td></td><td></td><td></td></tr>
<tr><td>N323 | H</td><td>T</td><td>T</td><td>1.6%</td><td>2.9%</td><td>6.9%</td></tr>
<tr><td>Q340 | H</td><td>F</td><td>F</td><td></td><td></td><td></td></tr>
<tr><td>Q345 | H</td><td>F</td><td>F</td><td></td><td></td><td></td></tr>
<tr><td>N359 | H</td><td>F</td><td>F</td><td></td><td></td><td></td></tr>
<tr><td>Q360 | H</td><td>F</td><td>F</td><td></td><td></td><td></td></tr>
<tr><td>N382 | H</td><td>T</td><td>T</td><td>17.9%</td><td>29.4%</td><td>44.9%</td></tr>
<tr><td>Q384 | H</td><td>F</td><td>F</td><td></td><td></td><td></td></tr>
<tr><td>N387 | H</td><td>T</td><td>T</td><td>23.3%</td><td>36.5%</td><td>43.0%</td></tr>
<tr><td>N388 | H</td><td>F</td><td>F</td><td></td><td></td><td></td></tr>
<tr><td>Q416 | H</td><td>F</td><td>F</td><td></td><td></td><td></td></tr>
<tr><td>Q417 | H</td><td>F</td><td>F</td><td></td><td></td><td></td></tr>
<tr><td>N419 | H</td><td>F</td><td>F</td><td></td><td></td><td></td></tr>
<tr><td>N431 | H</td><td>F</td><td>F</td><td></td><td></td><td></td></tr>
<tr><td>Q436 | H</td><td>F</td><td>F</td><td></td><td></td><td></td></tr>
<tr><td colspan="6">Antibody-2</td></tr>
<tr><td>Q3 | L</td><td>F</td><td>F</td><td></td><td></td><td></td></tr>
<tr><td>Q6 | L</td><td>F</td><td>F</td><td></td><td></td><td></td></tr>
<tr><td>Q24 | L</td><td>F</td><td>F</td><td></td><td></td><td></td></tr>
<tr><td>Q27 | L</td><td>F</td><td>F</td><td></td><td></td><td></td></tr>
<tr><td>N31 | L</td><td>F</td><td>F</td><td></td><td></td><td></td></tr>
<tr><td>N34 | L</td><td>F</td><td>F</td><td></td><td></td><td></td></tr>
<tr><td>Q37 | L</td><td>F</td><td>F</td><td></td><td></td><td></td></tr>
<tr><td>Q38 | L</td><td>F</td><td>F</td><td></td><td></td><td></td></tr>
<tr><td>Q79 | L</td><td>F</td><td>F</td><td></td><td></td><td></td></tr>
<tr><td>Q89 | L</td><td>F</td><td>F</td><td></td><td></td><td></td></tr>
<tr><td>Q90 | L</td><td>F</td><td>F</td><td></td><td></td><td></td></tr>
<tr><td>N93 | L</td><td>F</td><td>F</td><td></td><td></td><td></td></tr>
<tr><td>Q124 | L</td><td>F</td><td>F</td><td></td><td></td><td></td></tr>
<tr><td>N137 | L</td><td>F</td><td>F</td><td></td><td></td><td></td></tr>
<tr><td>N138 | L</td><td>F</td><td>F</td><td></td><td></td><td></td></tr>
<tr><td>Q147 | L</td><td>F</td><td>F</td><td></td><td></td><td></td></tr>
<tr><td>N152 | L</td><td>F</td><td>F</td><td></td><td></td><td></td></tr>
<tr><td>Q155 | L</td><td>F</td><td>F</td><td></td><td></td><td></td></tr>
<tr><td>N158 | L</td><td>F</td><td>F</td><td></td><td></td><td></td></tr>
<tr><td>Q160 | L</td><td>F</td><td>F</td><td></td><td></td><td></td></tr>
<tr><td>Q166 | L</td><td>F</td><td>F</td><td></td><td></td><td></td></tr>
<tr><td>Q199 | L</td><td>F</td><td>F</td><td></td><td></td><td></td></tr>
<tr><td>N210 | L</td><td>F</td><td>F</td><td></td><td></td><td></td></tr>
<tr><td>Q3 | H</td><td>F</td><td>F</td><td></td><td></td><td></td></tr>
<tr><td>Q13 | H</td><td>F</td><td>F</td><td></td><td></td><td></td></tr>
<tr><td>Q39 | H</td><td>F</td><td>F</td><td></td><td></td><td></td></tr>
<tr><td>Q57 | H</td><td>F</td><td>F</td><td></td><td></td><td></td></tr>
<tr><td>N73 | H</td><td>T*</td><td>T</td><td>4.4%</td><td>10.7%</td><td>26.6%</td></tr>
<tr><td>N76 | H</td><td>F</td><td>F</td><td></td><td></td><td></td></tr>
</table>

Q81 ǀ H	F	F			
N83 ǀ H	T	T	3.0%	5.3%	14.3%
N161 ǀ H	F	F			
Q177 ǀ H	F	F			
Q198 ǀ H	F	F			
N203 ǀ H	F	F			
N205 ǀ H	F	F			
N210 ǀ H	F	F			
N278 ǀ H	F	F			
N288 ǀ H	F	F			
Q297 ǀ H	F	F			
N299 ǀ H	F	F			
Q313 ǀ H	F	F			
N317 ǀ H	F	F			
N327 ǀ H	T	T	1.6%	3.0%	6.8%
Q344 ǀ H	F	F			
Q349 ǀ H	F	F			
N363 ǀ H	F	F			
Q364 ǀ H	F	F			
N386 ǀ H	T	T	17.7%	29.1%	45.2%
Q388 ǀ H	F	F			
N391 ǀ H	T	T	23.3%	36.5%	42.1%
N392 ǀ H	F	F			
Q420 ǀ H	F	F			
Q421 ǀ H	F	F			
N423 ǀ H	F	F			
N436 ǀ H	F	F			
Q440 ǀ H	F	F			
NISTmAb					
Q3 ǀ L	F	F			
Q6 ǀ L	F	F			
Q36 ǀ L	F	F			
Q37 ǀ L	F	F			
Q78 ǀ L	F	F			
Q89 ǀ L	F	F			
Q123 ǀ L	F	F			
N136 ǀ L	F	F			
N137 ǀ L	F	F			
Q146 ǀ L	F	F			
N151 ǀ L	F	F			
Q154 ǀ L	F	F			
N157 ǀ L	F	F			
Q159 ǀ L	F	F			
Q165 ǀ L	F	F			
Q198 ǀ L	F	F			
N209 ǀ L	F	F			
Q1 ǀ H	F	F			
Q16 ǀ H	F	F			
Q41 ǀ H	F	F			

N62 ǀ H	F	F			
N78 ǀ H	F	F			
Q79 ǀ H	F	F			
N86 ǀ H	F	F			
N104 ǀ H	F	F			
Q112 ǀ H	F	F			
N162 ǀ H	F	F			
Q178 ǀ H	F	F			
Q199 ǀ H	F	F			
N204 ǀ H	F	F			
N206 ǀ H	F	F			
N211 ǀ H	F	F			
N279 ǀ H	F	F			
Q298 ǀ H	F	F			
N300 ǀ H	F	F			
Q314 ǀ H	F	F			
N318 ǀ H	F	F			
N328 ǀ H	F	T	1.4%	2.9%	6.6%
Q345 ǀ H	F	F			
Q350 ǀ H	F	F			
N364 ǀ H	F	F			
Q365 ǀ H	F	F			
N387 ǀ H	T	T	17.6%	31.7%	43.2%
Q389 ǀ H	F	F			
N392 ǀ H	T	T	21.9%	41.5%	46.7%
N393 ǀ H	F	F			
Q421 ǀ H	F	F			
Q422 ǀ H	F	F			
N424 ǀ H	F	F			
N437 ǀ H	F	F			
Q441 ǀ H	F	F			

*In the initial trypsin digestion peptide mapping, this site was labeled as F (inactive) because no measurable deamidation was detected, owing to the loss of sequence coverage by the small tryptic peptide spanning over this residue; the label was corrected to T (active) upon experimental confirmation via LysC digestion peptide mapping which generated retained the sequence coverage.
